# Genetic Influences on Individual Differences in Exercise Behavior during Adolescence

**DOI:** 10.1155/2010/138345

**Published:** 2010-06-29

**Authors:** Niels van der Aa, Eco J. C. De Geus, Toos C. E. M. van Beijsterveldt, Dorret I. Boomsma, Meike Bartels

**Affiliations:** Department of Biological Psychology, VU University, Van der Boechorststraat 1, 1081 BT Amsterdam, The Netherlands

## Abstract

The aim of this study was to investigate the degree to which genetic and environmental influences affect variation in adolescent exercise behavior. Data on regular leisure time exercise activities were analyzed in 8,355 adolescent twins, from three-age cohorts (13-14, 15-16, and 17–19 years). Exercise behavior was assessed with survey items about type of regular leisure time exercise, frequency, and duration of the activities. Participants were classified as sedentary, regular exercisers, or vigorous exercisers. The prevalence of moderate exercise behavior declined from age 13 to 19 years with a parallel increase in prevalence of sedentary behavior, whereas the prevalence of vigorous exercise behavior remained constant across age cohorts. Variation in exercise behavior was analyzed with genetic structural equation modeling employing a liability threshold model. Variation was largely accounted for by genetic factors (72% to 85% of the variance was explained by genetic factors), whereas shared environmental factors only accounted for a substantial part of the variation in girls aged 13-14 years (46%). We hypothesize that genetic effects on exercise ability may explain the high heritability of exercise behavior in this phase of life.

## 1. Introduction

Regular exercise has been cited to be a key contributor to health [1], whereas a sedentary lifestyle is proposed to be one of the main causes of the rise in obesity that starts at an increasingly younger age [2]. Despite the well-documented benefits of exercise, many people do not exercise on a regular basis [3]. As a consequence, a sedentary lifestyle, and the accompanying risk for obesity, remains a major threat to health in today's society. Studying exercise behavior during adolescence is of particular interest because several studies reported that the prevalence of exercise participation declines with increasing age, and that this decline is most prominent during adolescence [4–6]. 

To increase the success of intervention on this important health-related behavior, much research has been devoted to the determinants of exercise behavior. The main focus of these studies has been on social, demographic, and environmental characteristics, such as low socioeconomic status and low social support by family and peers [7–9]. None of these factors, however, have emerged as a strong causal determinant of exercise behavior, with the possible exception of gender, showing that exercise participation is higher in boys than it is in girls. Twin studies offer the possibility to assess the importance of genetic factors as determinants of exercise behavior. With data from twins, individual differences in behavior can be decomposed as due to genetic, shared environmental (environmental influences shared by members of the same family) and nonshared environmental influences (influences unique to an individual). The importance of genetic and environmental factors can be estimated by comparing the resemblance in exercise behavior between monozygotic (MZ) twins and dizygotic (DZ) twins. A greater resemblance of MZ twins, who are genetically identical, compared to DZ twins, who share on average half of their segregating genes, constitutes evidence for genetic influences on exercise behavior. If MZ twins resemble each other more than DZ twins, but not to the extent that would be expected based on their twice larger genetic resemblance, shared familial factors may also be important [10]. 

A number of twin studies have shown that genetic factors contribute to individual differences in exercise behavior and measures of exercise frequency, duration, and intensity during adolescence and adulthood [4, 11–15]. The genetic architecture of exercise behavior has been found to differ across the life span with the largest differences seen during adolescence [16]. In a Dutch twin study, Stubbe et al. [4] found that genetic variation was of no importance to leisure time exercise in 13- to 16-year-old adolescents. Instead, environmental influences shared by siblings from the same family accounted for the largest part of variation in exercise behavior. From age 17 the role of shared environmental influences rapidly waned and genetic influences started to dominate the individual variation in exercise behavior. A combination of genetic and shared environmental influences on exercise behavior in adolescence has also been reported by other studies [11, 12]. In contrast to Stubbe et al. [4] who found no difference in the genetic architecture between boys and girls, these studies suggested clear sex differences such that the shared environment lost its importance earlier in boys than in girls. In part, the discrepancies in the sex-specific genetic architecture across these previous studies may reflect insufficient statistical power to reliably detect age by sex effects.

 In the present study, we examined the relative influence of genetic and environmental factors on self-reported leisure time exercise behavior in the largest sample of adolescent twins to date. Due to the large sample size this study was able to estimate genetic and environmental influences within three different age groups (13-14, 15-16, and 17–19 years) and to assess quantitative sex differences (e.g., differences in heritability) as well as qualitative sex differences (are the same genes expressed in boys and girls) in the genetic architecture within these age groups.

## 2. Methods

### 2.1. Subjects

Participants were registered with the Netherlands Twin Registry (NTR), established by the Department of Biological Psychology at the VU University in Amsterdam [17, 18]. The large majority of twins had been registered with the NTR as newborns. Parents of adolescent twins were asked for consent to send their children a survey. If their parents consented, twins and their nontwin siblings received an online or a paper and pencil self-report survey when they were 14, 16, and 18 years. The survey contained items about behavior, sport, lifestyle, and well-being. When twins and siblings did not return the survey on time they were contacted by mail for a first reminder and next they were contacted by phone for a second reminder. A total of 3,645 families with twins born between 1986 and 1994 participated in this ongoing study at least once so far. The overall family response rate is 56%. 

Triplets and nontwin siblings were not included in the present paper. Furthermore, twins with an illness or handicap interfering with their daily lives were also not included. This resulted in a total sample of 8,355 twins (42% male) from complete and incomplete pairs, coming from 3,405 families. For 1,160 twins, data were available at two time points. Participants were primarily Caucasian and they came from all regions of The Netherlands (rural and urban areas). Data were available for 754 (17%) incomplete and 3,614 (83%) complete twin pairs. In Table 1, zygosity of the participating twin pairs is presented. For 1,089 (36.1%) of the same-sex twin pairs zygosity was determined based on blood group or DNA typing. Zygosity for the remaining same-sex twin pairs was determined by questionnaire items about physical similarities and confusion by family members and strangers. These items allow accurate determination of zygosity in 93% of same-sex twin pairs [19].

 Participants were divided into the age groups 13-14 years (33%), 15-16 years (38%), and 17–19 years (29%). Mean age in the three-age groups was 14.51 years (SD = 0.31), 16.23 (SD = 0.61), and 18.06 (SD = 0.70), respectively. The age groups were not completely independent, because for a small subset of participants data were available at two time points (e.g., twins returned a survey at age 14 and 16). Furthermore, since a small subset of participants participated in a pilot and short there after in the regular survey collection data from 2 surveys were present within one age group. For this subset of participants, data from the pilot version were excluded for the analyses. As can be seen in Table 1, each age group had adequate numbers of monozygotic (MZ) and dizygotic (DZ) twin pairs.

### 2.2. Exercise Behavior

Participants were asked to indicate what type(s) of regular leisure exercise they were involved in at the time of assessment. A list of 21 common individual (includes fitness centre, jogging, tennis, etc.) and team-based exercise activities (soccer, field hockey) was provided plus 5 open entries for less common activities. For each exercise activity endorsed, the participants further reported how many months per year, weekly frequency and the average duration of the activitiy. Ainsworth's Compendium of physical activity [20] was used to assign an MET score (Metabolic equivalent) to each exercise activity, reflecting its energy expenditure as a multiple of the basal energy expenditure (approximately 1 kcal/kg/hour) in an average subject engaged in that activity. When in high-school, Dutch adolescents have to attain physical education (PE) classes for 1–3 hours per week. The exact amount of MET hours weekly in these PE classes was assessed as a separate variable. 

 For each participant a total weekly MET score was computed across all exercise activities by summing the products of the number of hours spent weekly on each exercise activity and its MET score. Activities were only scored if that participants had engaged in them for at least three months during the past year. Exercise during physical education classes at school was not included in the weekly MET score. Thus the dependent variable reflects leisure time exercise behavior only.

 Participants were classified into three groups based on their total MET scores. The first category consisted of sedentary participants whose total weekly MET score was lower than 5.0. The second category of moderate exercisers consisted of participants whose total weekly MET score ranged between 5.0 and 30.0. The third category consisted of vigorous exercisers whose total weekly MET score was 30.0 or higher.

### 2.3. Statistical Analyses

Because the data of exercise behavior were positively skewed, a liability threshold model was used to analyze individual differences in exercise behavior within each age group. The basic assumption underlying the liability threshold model, which was originally proposed by Falconer [21], is that an unobserved (latent) continuous liability underlies the skewed distribution of the observed variable (i.e., exercise behavior). The liability is assumed to be standard normal distributed (i.e., mean = 0, SD = 1). With family data, correlations between family members can be estimated for the liability, rather than for the observed trait. After obtaining the correlations between twins for the liability distribution, we employed genetic structural equation modeling to estimate the relative contributions of genetic and environmental influences to individual differences in liability to exercise behavior.

**Figure 1:**
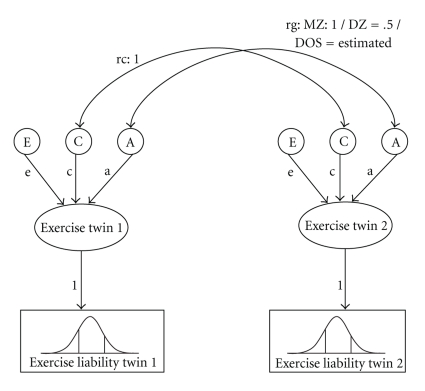
Univariate liability threshold model for twin data. Exercise behavior was measured with 3 categories (hence 2 thresholds are estimated). The total variance in liability is one and is modeled as caused by latent factors A (additive genetic influences), C (common or shared environment) and E (unique environment). The square of path coefficients a, c, and e gives the variance due to A, C and E.

 Participants were classified into three groups (i.e., sedentary, moderate exercise, vigorous exercise) as described above. In this way, ordinal scores on exercise behavior were obtained that were coded 0, 1, and 2. To model the three categories of exercise behavior two thresholds were required. The thresholds, expressed in *z*-values, are defined by the prevalence of the three categories of exercise behavior in the sample and represent the value in the latent liability distribution above which an individual will endorse the next category. In the lower part of Figure 1, the distribution of exercise liability is presented. As can be seen in the figure, the thresholds represented by the vertical lines, separate the exercise liability distribution into three distinct categories.

Resemblance in the liability to exercise behavior between twins is expressed in polychoric twin correlations. Comparing MZ twin correlations with DZ twin correlations provides a first step in evaluating the relative influence of genetic and environmental factors on individual differences in liability to exercise behavior. When the MZ correlation is higher than the DZ correlation, it is inferred that genetic variation influences individual differences in liability to exercise behavior. A DZ correlation higher than half the MZ correlation implies shared environmental effects, referring to environmental factors shared by all members of the same family, on liability to exercise behavior. Variation that is not due to genetic and shared environmental effects is attributed to environmental effects which are not shared by family members. The nonshared environmental variance component also includes measurement error variance. Comparing MZ and DZ twin correlations in boys and girls provides specific information regarding quantitative sex differences. When the difference between MZ and DZ twin correlations is larger in boys than in girls, it can be concluded that genetic influences are stronger in boys compared to girls. Specific information regarding qualitative sex differences can be derived from the DZ opposite-sex (DOS) correlation. When the twin correlation in DOS twin pairs is lower than predicted from the correlation in DZ-male and DZ-female twin pairs this might be due to genetic or shared environmental effects that influence one sex but not the other [21].

 As a first step, the thresholds and polychoric twin correlations were estimated for each of the 5 sex by zygosity groups (i.e., MZM, DZM, MZF, DZF, and DOS) using the software package Mx [22]. Thresholds were estimated separately for boys and girls to take into account sex differences in the prevalence of exercise behavior. This model is referred to as a saturated model and simply specifies for each sex by zygosity group that the data from the first- and second-born twin are correlated without attempting to model these correlations as a function of genes or shared environment. Within a series of nested models we tested whether constraining the thresholds to be equal between boys and girls led to a significant deterioration of model fit. In addition, we tested whether twin correlations were significantly different for MZ and DZ twins.

 Next, genetic models were fitted to the data in which the genetic architecture of liability to exercise behavior was specified for each age group. A graphical representation of the genetic model is given in Figure 1. The amount of variance in the underlying liability due to additive genetic (A), shared environmental (C), and nonshared environmental effects (E) can be estimated by considering the different level of genetic relatedness between MZ and DZ twin pairs. MZ twin pairs are genetically identical, whereas DZ twin pairs share on average 50% of their segregating genes. In the genetic models, the genetic correlation (rg) for MZ and DZ twin pairs is therefore fixed at 1.0 and 0.5 respectively. Shared environmental effects refer to environmental factors that are shared by all siblings in the family and therefore the shared environmental correlation (rc) is fixed at 1.0. In Figure 1, rg and rc are represented by the double-headed arrows connecting the latent genetic (A) and shared environmental factors (C) of both members of a twin pair. Nonshared environmental effects refer to environmental factors that are unique to individuals in the family and therefore these are uncorrelated between siblings. The influence of A, C, and E is represented by path coefficients *a*, *c,* and *e* (see Figure 1). Because it is assumed that the liability to exercise is standard normal distributed, the total variance under the liability distribution is 1. The influence of A, C, and E therefore also adds up to 1. Under this model, proportions of variance explained by genetic, shared environmental, and nonshared environmental factors can be obtained by squaring the corresponding path coefficients *a, c, *and* e*.

Ifqualitative sex differences in liability to exercise behavior are present, the genetic correlation for DOS twins should be lower than the genetic correlation for DZ twins. To assess qualitative sex differences the genetic correlation (rg) between DOS twins was estimated and we tested whether fixing rg to 0.5 resulted in a significant deterioration of model fit. Quantitative sex differences in liability to exercise behavior were assessed by allowing the genetic (*a*), shared environmental (*c*), and nonshared environmental (*e*) parameter estimates to differ for boys and girls and we tested whether constraining these parameter estimates to be equal for boys and girls resulted in a significant deterioration of model fit. The statistical significance of the variance components A and C was assessed by testing whether fixing the corresponding parameter estimate (i.e., *a* and *c*) to zero resulted in a significant deterioration of model fit. 

 We fitted various models that were nested in the sense that one model could be derived from the other by the imposition of one or more constraints on the parameters. The fit of the different models was compared by means of the log-likelihood ratio test (LRT). The difference in minus two times the log-likelihood (-2LL) between two nested models has a *χ* ^2^ distribution with the degrees of freedom (df) equaling the difference in df between the two models. If a *P*-value higher than 0.05 was obtained from the *χ* ^2^-test the fit of the constrained model was not significantly worse than the fit of the more complex model. In this case, the constrained model was kept as the most parsimonious and best fitting model. The fit of the genetic models was also compared to the full ACE model by means of Akaike's Information Criterion, keeping the model with the lowest AIC as the best fitting model [22].

## 3. Results

Table 2 presents the prevalence of exercise behavior for the three-age groups. The table shows that irrespective of age, sedentariness and moderate exercise behavior are more prevalent in girls, whereas vigorous exercise behavior is more prevalent in boys. Formal tests on the thresholds showed these differences to be significant. The thresholds were different between boys and girls in the 13-14 years (*χ* ^2^(2) = 87.44, *P* < .01), 15-16 years (*χ* ^2^(2) = 84.33, *P* < .01), and the 17–19 years (*χ* ^2^(2) = 64.03, *P* < .01) age groups. In boys and girls, there is an increase in the prevalence of sedentariness (i.e., decrease in exercise behavior) in the 17–19 olds compared to the other age groups whereas there is a parallel decrease in the prevalence of moderate exercise behavior. The prevalence of vigorous exercise behavior remains constant throughout adolescence.

Twin correlations in the different age groups are presented in Table 3. For boys and girls, MZ twin correlations were significantly higher than DZ twin correlations in the 13-14 years (*χ* ^2^(2) = 63.20, *P* < .01), 15-16 years (*χ* ^2^(2) = 43.09, *P* < .01), and 17–19 years (*χ* ^2^(2) = 23.94, *P* < .01) age groups, suggesting that individual differences in liability to exercise behavior are influenced by genetic factors. For girls in the youngest age group, resemblance in exercise behavior between MZ twins was similar to DZ twins, suggesting that shared environmental factors play an important role in explaining individual differences in exercise behavior. For girls in the two oldest age groups and for boys in all age groups DZ twin correlations were about half the MZ twin correlation, suggesting that genetic factors explain the bulk of variation in exercise behavior for these age groups. 

 Genetic model fitting results for all age groups are presented in Table 4. In model 2, rg was constrained at 0.5 which did not result in a significant deterioration of model fit in any of the three-age groups, indicating that the same genetic factors act in boys and girls with regard to exercise behavior. 

Model 3 tested whether constraining the parameter estimates of the full univariate ACE model to be equal for boys and girls led to a significant deterioration of model fit. In the youngest age group there appeared to be significant differences in the magnitude of the variance components explaining individual differences in liability to exercise behavior. Therefore, parameter estimates were allowed to differ between boys and girls for this age group. In the two oldest age groups constraining the parameter estimates to be equal between boys and girls did not lead to a significant deterioration of model fit. 

Models 4 and 5, tested whether constraining the genetic or shared environmental parameter estimate to zero would lead to a significant deterioration of model fit. Additive genetic effects on individual differences in liability to exercise behavior were statistically significant in all age groups. Shared environmental effects were statistically significant for girls in the youngest age group. In all age groups the LRT tests and the AIC pointed to the AE model as the most parsimonious model, except for girls in the youngest age group in which the ACE model was most parsimonious. 

The proportions of variance explained by A, C, and E in liability to exercise behavior of the three-age groups are summarized in Table 5. For boys in all age groups, the proportion of variation in liability to exercise behavior explained by genetic factors ranged between  .72 and  .85. The remaining variation was accounted for by nonshared environmental factors. For girls in the youngest age group, genetic and shared environmental factors accounted for individual differences in exercise behavior, .38 and  .46 respectively. For girls in the two oldest age groups, shared environmental factors did not account for variation in liability to exercise behavior, whereas the proportions of liability explained by genetic factors were  .80 and  .72.

## 4. Discussion

In a large sample of Dutch adolescent twins, we found that the prevalence of sedentariness increased during late adolescence compared to early adolescence. At all ages, girls were more often sedentary than boys. When regularly engaged in exercise, girls more often exercised at a moderate rather than a vigorous level. During early adolescence, individual differences in liability to exercise behavior could be accounted for by genetic and nonshared environmental factors for boys, whereas for girls shared environmental factors accounted for a substantial part of the individual differences as well. During middle and late adolescence, genetic influences accounted for the largest part of the variation in liability to exercise behavior for boys as well as girls. No evidence was found for qualitative sex differences in the genetic factors, indicating that the same genetic variants appear to influence exercise behavior in boys and girls.

Our finding that the prevalence of moderate exercise behavior decreased during late adolescence in boys and girls in favor of the prevalence of sedentariness corresponds with the results of other studies [4–6]. The prevalence of vigorous exercise, however, did not change across the three-age groups. This finding is consistent with Van Mechelen et al. [6] who observed a graduate decline in the prevalence of physical activities of mild intensity and nonorganized sports activities, but not in the prevalence of organized sports activities. An explanation for this is that vigorous exercisers have strong intrinsic motivations to exercise leading to continuation of their exercise behavior, whereas moderate exercisers are less intrinsically motivated to exercise making them more likely to become sedentary.

The main aim of the present study was to assess to what extent genetic and environmental factors affect exercise behavior from early to late adolescence. For boys, genetic factors accounted for the major part of individual differences in exercise behavior from early to late adolescence. It has been suggested that genetic influences on exercise ability may explain part of the heritability of exercise behavior [23, 24]. The basic idea is that people will seek out the activities that they are good in. This is particularly true in male adolescents, because being good in sports is an important source of self-esteem for these adolescents and the athletic role model is continuously reinforced by the media [25, 26]. Therefore, genes coding for exercise ability (endurance, strength, flexibility, motor coordination) may well become genes for adolescent exercise behavior. 

In contrast to boys of the same age, shared environmental factors accounted for a major part of individual differences in exercise behavior for the youngest girls, whereas from 15 years onwards the influence of these shared environmental factors had completely disappeared in favor of genetic factors. Shared environmental influences may include parents, siblings and peers who make sure the young adolescent girls regularly get to the playing field, and to provide positive feedback on their performance. The extent of positive feedback from parents, siblings and especially from peers may increasingly depend on their genotypes for exercise ability. In short, the shared environment determines exposure and encouragement in early adolescence, but, as for the boys, actual exercise ability will determine whether girls like exercising enough (by excelling in it) to maintain the behavior when the perception of peers increases in relative importance to that of parents during mid and late adolescence. The idea that a single factor like exercise ability is crucial to both boys and girls is reinforced by the fact that the same qualitative genetic variation was seen to underlie the heritability of exercise behavior in boys and girls.

The genetic architecture of exercise behavior during adolescence has been addressed in previous studies [4, 11, 12]. In a sample of the Netherlands Twin Registry from an earlier birth cohort, Stubbe et al. [4] also found a shift from shared environmental to genetic influences during adolescence. However, they reported the shift to occur during late adolescence (i.e., around 16 years) and shared environmental effects on exercise behavior were found not only for young adolescent girls but also for the boys. The sample had very similar age groups as in the present study but the data were collected 10 to 15 years earlier, that is in a birth cohort born 10 to 15 years earlier than the current cohort. The much larger sample size of the present study and its more extensive assessment of leisure time exercise behavior may have led to increased precision of the estimated parameters. 

Additional support for the pattern of sex differences in the genetic architecture of exercise behavior in adolescents found in the present study comes from other studies in different countries. In a small Flemish sample of 15 year-old twins Beunen and Thomis [12] found that 83% and 44% of variability in exercise behavior is accounted for by genetic factors for boys and girls respectively, and 54% is accounted for by shared environmental factors only in girls. In a study based on 411 Portuguese twins aged 12–25 years, Maia et al. [11] found larger heritability estimates for boys (68%) compared to girls (40%). Unfortunately, both studies were too small to divide their samples into different age cohorts and it could not be established whether the sex differences were specific to certain age groups.

A limitation of the present study was the use of a cross-sectional twin design to examine the relative influence of genetic and environmental influences on individual differences in exercise behavior. The genetic architecture of exercise behavior during adolescence is most properly addressed in a longitudinal design. So far data at two time points are only available for a small subsample, and data throughout adolescence (13–18) are absent. Since our data collection is a continuous process at the NTR we anticipate large enough longitudinal sample size within the next 5 years. Large shifts in the genetic architecture are expected when subjects move from adolescence to adulthood. In adulthood, nonshared environmental factors become more important and heritability decrease to about 50% [23]. Furthermore significant qualitative sex differences are found in adulthood with different genetic factors influencing male and female exercise behavior [23, 27].

## 5. Conclusions

The prevalence of moderate exercise behavior declined from age 13 to 19, whereas the prevalence of vigorous exercise behavior remained constant across age groups. Variation in exercise behavior could be largely accounted for by genetic factors, whereas shared environmental factors only accounted for a substantial part of the variation in girls aged 13-14 years. Future studies should focus on the role of exercise ability as a potential determinant of exercise behavior. If the high heritability of exercise behavior in this phase of life is indeed explained by genetic effects on exercise ability—a testable hypothesis—then the relatively high levels of sedentary adolescents may reflect an undesirable emphasis on performance rather than pleasure in current day adolescent sports culture.

## Figures and Tables

**Table 1:** Zygosity of participating twin pairs for the total sample and the different age groups (complete twin pairs added in parentheses).

	Total sample	13-14 yr	15-16 yr	17–19 yr
MZM	662 (585)	211 (197)	282 (249)	169 (139)
DZM	567 (465)	201 (170)	210 (184)	156 (111)
MZF	1042 (918)	343 (317)	380 (333)	319 (268)
DZF	738 (621)	231 (207)	265 (225)	242 (189)
DOS	1359 (1025)	516 (428)	494 (372)	349 (225)

Note. MZM: monozygotic male twin pair; DZM: dizygotic male twin pair; MZF: monozygotic female twin pair; DZF: dizygotic female twin pair; DOS: dizygotic opposite-sex twin pair.

**Table 2:** Prevalence (95% confidence intervals between parentheses) of exercise participation in the different age groups as a function of sex.

	13-14 yr		15-16 yr		17–19 yr	
	Boys	Girls	Boys	Girls	Boys	Girls
Sedentariness	20% (17%–22%)	31% (28%–33%)	24% (21%–26%)	31% (29%–34%)	27% (24%–31%)	38% (35%–40%)
Moderate exercise	40% (39%–41%)	45% (45%–46%)	35% (35%–36%)	45% (44%–45%)	31% (30%–31%)	38% (38%–39%)
Vigorous exercise	40% (37%–44%)	24% (21%–26%)	41% (38%–44%)	24% (21%–27%)	41% (38%–46%)	24% (21%–27%)

**Table 3:** Twin correlations for exercise participation in each age group (95% confidence intervals added in parentheses).

	13-14 yr	15-16 yr	17–19 yr
MZM	.85 (.79–.90)	.76 (.67–.82)	.73 (.60–.82)
DZM	.23 (.01–.42)	.48 (.32–.62)	.48 (.27–.65)
MZF	.83 (.78–.88)	.83 (.77–.87)	.71 (.63–.78)
DZF	.67 (.56–.75)	.52 (.39–.63)	.34 (.15–.50)
DOS	.32 (.21–.42)	.36 (.25–.47)	.29 (.12–.44)

**Table 4:** Univariate model fitting results for exercise behavior in the three-age groups.

Model	vs	-2LL	df	*χ* ^2^	Δdf	*P*	AIC
*13-14* *yr *							
(1) ACE: sex differences (rg estimated)	—	5482.577	2812	—	—	—	—
(2) ACE: sex differences (rg fixed at 0.5)	1	5482.704	2813	0.127	1	.72	−1.87
(3) ACE: no sex differences (rg fixed at 0.5)	2	5502.510	2815	19.81	2	<.01	13.93
(4)^(a)^ CE: boys, ACE: girls (rg fixed at 0.5)	2	5536.641	2814	53.94	1	<.01	50.06
(4)^(b)^ ACE: boys, CE: girls (rg fixed at 0.5)	2	5497.697	2814	14.99	1	<.01	11.12
(5)^(a)^ *AE: boys, ACE: girls (rg fixed at 0.5) *	**2**	5483.223	2814	0.52	1	.47	−3.35
(5)^(b)^ACE: boys, AE: girls (rg fixed at 0.5)	2	5502.504	2814	19.80	1	<.01	15.93

*15-16* *yr *							
(1) ACE: sex differences (rg estimated)	—	5943.005	2986	—	—	—	—
(2) ACE: sex differences (rg fixed at 0.5)	1	5944.573	2987	1.57	1	.21	−.43
(3) ACE: no sex differences (rg fixed at 0.5)	2	5949.728	2989	5.16	2	.08	.72
(4) CE: no sex differences (rg fixed at 0.5)	3	6024.535	2990	74.81	1	<.01	73.53
(5) *AE: no sex differences (rg fixed at 0.5) *	**3**	5950.674	2990	.95	1	.33	−2.33

*17–19* *yr *							
(1) ACE: sex differences (rg estimated)	—	4455.979	2158	—	—	—	—
(2) ACE: sex differences (rg fixed at 0.5)	1	4455.979	2159	.00	1	>.99	−2.00
(3) ACE: no sex differences (rg fixed at 0.5)	2	4458.120	2161	2.14	2	.34	−3.86
(4) CE: no sex differences (rg fixed at 0.5)	3	4495.737	2162	37.62	1	<.01	21.76
(5) *AE: no sex differences (rg fixed at 0.5) *	**3**	4458.120	2162	.00	1	>**.99**	−5.86

Note. vs: versus; -2LL: −2log  likelihood; df = degrees of freedom; *χ* ^2^ = chi-square test statistic; Δdf = degrees of freedom of *χ* ^2^ test; *P* = *P*-value; AIC = Akaike's Information Criterion; rg = genetic correlation between DOS twins. Most parsimonious models are printed in boldface type.

**Table 5:** Proportions of variance explained by additive genetic, common environmental and unique environmental factors from the best-fitting models for exercise participation in three-age groups for boys and girls (95% confidence intervals added in parentheses).

		A	C	E
13-14 yr	Boys	.85 (.78–.90)	—	.15 (.10–.22)
	Girls	.38 (.22–.57)	.46 (.27–.61)	.16 (.12–.21)
15-16 yr	Boys	.80 (.76–.84)	—	.20 (.16–.24)
	Girls	.80 (.76–.84)	—	.20 (.16–.24)
17–19 yr	Boys	.72 (.65–.77)	—	.28 (.23–.35)
	Girls	.72 (.65–.77)	—	.28 (.23–.35)
